# Topics for implementation research: Implementation researchers’ and practitioners’ views in The Netherlands

**DOI:** 10.1186/s43058-026-00890-6

**Published:** 2026-02-24

**Authors:** Femke van Nassau, Anouk Driessen, Leti van Bodegom-Vos, Bethany Hipple Walters, Erwin Ista, Wouter Keijser, Rianne van der Kleij – van der Sluis, Gera Welker, Michel Wensing, Christiaan Vis

**Affiliations:** 1 Department of Public and Occupational Health, Amsterdam Public Health research institute, Amsterdam UMC location Vrije Universiteit Amsterdam, Amsterdam, the Netherlands; 2 Department of Biomedical Data Sciences, Leiden University Medical Center, Leiden, the Netherlands; 3 Centre for Implementation, the Trimbos Institute, Utrecht, The Netherlands; 4 Department of Internal Medicine, Division Nursing Science, Erasmus MC, Erasmus University Medical Center, Rotterdam, The Netherlands; 5 DIRMI Institute, Utrecht, The Netherlands; 6 Department of Public Health & Primary Care, Leiden University Medical Center, Leiden, the Netherlands; 7 UMC Staff Policy and Management Support, University Medical Center Groningen, University of Groningen, Groningen, The Netherlands; 8 Department of General Practice and Health Services Research, Heidelberg University Hospital, Im Neuenheimer Feld 130.3, Heidelberg, Germany

**Keywords:** Implementation research, Implementation practice, Agenda setting, e-Delphi

## Abstract

**Background:**

In recent years, implementation research has gained a renewed attention in the Netherlands. However, limited national funding for implementation research has mainly resulted in case- and context-specific descriptive data. To help prioritize research that holds high scientific value and practical relevance, this study aimed to identify gaps in both implementation science and implementation practice.

**Methods:**

A two-stage study was conducted combining multiple methods to collect data from implementation researchers working in the healthcare sector in the Netherlands. A two-round e-Delphi study was employed to identify research priorities amongst implementation researchers. In addition, a survey was conducted with practitioners to identify implementation knowledge gaps and needs in implementation practice.

**Results:**

Twenty-six (55%) of the 47 invited researchers participated in Round 1 of the e-Delphi, leading to the identification of 31 research topics categorized into 7 themes. In Round 2, 22 of the 26 researchers (85%) completed the process, reaching consensus on 12 topics. These topics were grouped into six themes and linked to four areas of research: implementation, sustainability, scale-up, and de-implementation. The themes include: (1) understanding determinants, (2) matching strategies to determinants, (3) implementation strategies, (4) measuring implementation outcomes, (5) theories, models and frameworks, and (6) research designs. The survey of 74 practitioners revealed 230 implementation knowledge gaps, which were then triangulated with the e-Delphi results, highlighting specific research topics that emphasize implementation capacity and the need for pragmatic tools to enhance evidence-based implementation in practice.

**Conclusions:**

By integrating insights from both implementation researchers and practitioners, the research agenda addresses topics that are relevant to both fields. Recommendations were made to advance the scientific field and improve implementation practice. This research agenda can guide research coordination and policymaking, aiming to consolidate research efforts in the Netherlands.

**Supplementary Information:**

The online version contains supplementary material available at 10.1186/s43058-026-00890-6.

Contributions to the literature

- This Dutch agenda is the first to combine perspectives from both implementation researchers and practitioners unlike existing agendas which are mostly expert-based and focus on researchers’ views.
- This study highlights a shift in research priorities from merely identifying determinants to focusing on the mechanisms and effectiveness of implementation strategies and paying attention to the dynamics in different phases of implementation work (pre-, implementation, sustainment, scaling-up, de-implementation).
- This agenda provides researchers, practitioners, policy makers and funders with clear directions and encourages them to use it as a framework to align their focus and priorities in implementation research.

## Background

Since the end of World War II, pioneers in implementation science, knowledge transfer, and healthcare quality improvement have been recognized. In 2006, the journal Implementation Science was launched to serve the scientific community, offering a dedicated platform for discussing and sharing knowledge on improving implementation methods and outcomes [1, 2]. Since that time, the funding and dissemination of exploratory, fundamental, practice-oriented implementation research has grown substantially. Many landmark papers have been published, culminating in a variety of theories, models, frameworks, taxonomies, guidelines, and research agendas [3].

The field has evolved from its early focus on describing and defining implementation challenges to investigating the causal pathways that influence the effectiveness of implementation efforts [4]. Many other relevant aspects have emerged over time, including issues of sustaining recently implemented interventions in practice, scaling-up evidence-based interventions to reach population level impacts, and de-implementation of ineffective or inefficient current healthcare practices.

In 2009, Eccles and colleagues [1] presented an overview of research needs. Similarly in 2012, the National Institutes of Health in the United States issued a report providing future directions for implementation research in United States healthcare settings [5]. Other agendas are focussed on specific medical conditions or intervention types such as HIV [6, 7], occupational therapy profession [8], or for specific target populations such as the elderly [9], children [10, 11], mental health peer specialists [12], or in specific settings such as schools [13], medical educational settings [14], global health settings [15], and public health [9]. In addition, research agendas have been published for specific implementation research topics, such as implementation strategies [16], implementation outcomes [17], sustainability [18], scale-up [19], and cost-effectiveness [20]. Despite the applied nature of the field [21] and the relevance of context [22], these agendas miss addressing the needs of implementation practice. Existing agendas are mostly expert-based and predominantly take the researchers’ perspective into account.

Implementation research in the Netherlands emerged as an applied field in the 1990s. It’s emergence was driven by growing concerns regarding knowledge utilisation that became embedded within Dutch health research funding and policy frameworks, notably through ZonMw’s policy on knowledge utilisation. Up to 2010, Dutch implementation research in the Netherlands was largely practice-driven, focusing on the identification and classification of determinants and strategies, the synthesis of existing evidence, and the development of theories, models, and frameworks to guide implementation efforts alongside and structure implementation problems [3, 23, 24]. By the early 2020s, implementation research had entered a second phase, with increased attention to studies of implementation effectiveness in a broad range of fields, following international trends [17, 24–26], and had evolved into an organised domain in the Netherlands marked by the establishment of dedicated research centres, national networks, regular symposia, formal training programmes, and a growing emphasis value-driven de-implementation, and system-level implementation approaches for scale up [27].

During this period, implementation science in the Netherlands transitioned from predominantly ad hoc, project-based activities only to include attempts to develop a more structured, institutionalised, and strategically oriented field [28]. Nevertheless, linear knowledge-to-practice thinking models, and limited attention for context dependency, and system complexity continued to be widely present despite the growth of the field, evaluation of implementation programs still not always take place, and most evaluations have a descriptive and non-generalizable nature [29]. At the same time, (financial) national resources for implementation research remain scarce and scattered [28].

In the broader context, the Netherlands hosts a distinctive setting for implementation science due to the organisation of its health system and research infrastructure. Healthcare is delivered through a system of regulated competition, with universal insurance coverage and a strong primary care sector in which general practitioners act as gatekeepers to specialist services. Moreover, health research funding is highly centralised, with ZonMw functioning as the dominant public funder and intermediary between government, academia, and practice (www.ZonMw.nl). This enables relatively strong national steering of research agendas, while also fostering a culture of programme-based and policy-oriented research.

To contribute to systematically prioritising implementation research in the Netherlands that is of high scientific value and practically relevant, we sought to answer the following main research question: What are current knowledge gaps in implementation research and practice? Subsequently, the article also contains recommendations for advancing the scientific field and enhancing implementation practice in the Netherlands.

Because the contextual features healthcare systems are likely to influence the practical relevance of the outcomes of an implementation science research agenda-setting exercise, we focussed the study on the Dutch healthcare and implementation context. The scope of this study and its findings should therefore be critically viewed in light of a pluralistic, decentralised healthcare service landscape with funding centralisation, and specific innovation and governance arrangements, which likely condition the necessity and feasibility of implementation research priorities.

## Methods

**Fig. 1:**
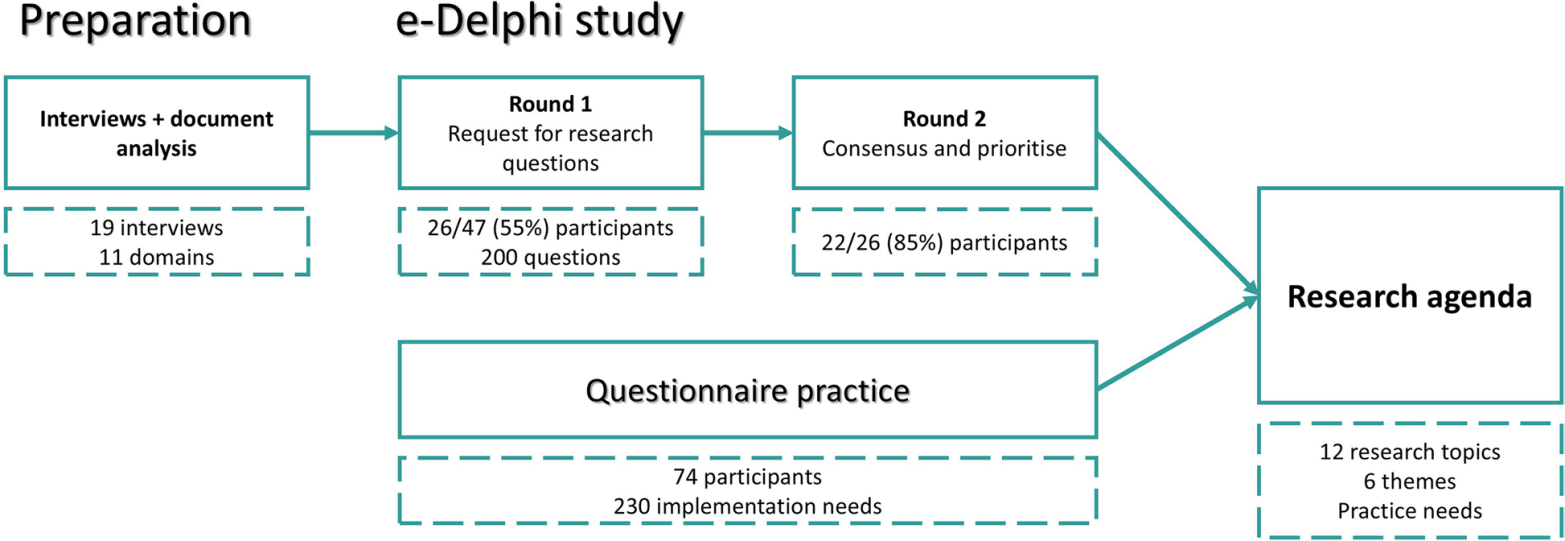
A two-stage study design incorporating perspectives of both implementation researchers and practitioners working in healthcare in the Netherlands

A two-stage study design was applied combining an e-Delphi study [30] with a survey. The e-Delphi study was conducted among implementation researchers working in the Dutch healthcare system and aimed to identify relevant implementation research topics. Next, we verified the results using a survey to identify knowledge gaps in implementation practice and further inform the list of topics. The survey was conducted with practitioners involved in implementation processes in the Dutch healthcare system. An overview of the methods used is provided in Fig. 1.

Throughout the study we adhered to the following definition of implementation research: “*the scientific study of methods to promote the systematic uptake of research findings and other evidence-based practices into routine practice, and, hence, to improve the quality and effectiveness of health services. It includes the study of influences on healthcare professional and organisational behaviour*” [31].

In the survey, we framed implementation practice following the European Implementation Collaboratives definition on its site*: “Implementation refers to a set of planned, intentional activities that aim to put into practice evidence-informed policies and practices in real-world services.”* (www.implementation.eu).

The study was conducted between July 2021 and March 2022. The Medical Ethics Review Committee of VU University Medical Center confirmed that the Medical Research Involving Human Subjects Act (WMO) does not apply to this study and approval was not required (registration number 2021.0432). All participants provided written informed consent.

### Steering group

We formed a steering group to guide the development of the study methodology and interpretation. This group consisted of at that time members of the Netherlands Implementation Collective (NIC) working group on Implementation Research, who are authors on this paper and are experienced in the field of implementation science. This group provided feedback on the interview topic list, the rounds of the e-Delphi study, and analysis of the data. This group did not participate in the e-Delphi study.

### Preparation

The e-Delphi study consisted of two rounds and was prepared with information from semi-structured interviews with selected key informants and a quick-scan of relevant documents, such as national funding evaluation reports, existing Dutch knowledge agenda (e.g. prevention agenda), report of expert meetings. A topic list was developed that was used in semi-structured interviews with implementation researchers in the Netherlands. Interviewees were recruited amongst members of the NIC and through snowball sampling. Implementation researchers were eligible to participate in the interviews when they had published on implementation focussed research. The interviews (FN and AD) lasted for 60 min and were held in Dutch. In total, 19 implementation researchers in the Netherlands participated in the semi-structured interviews. Participants conducted implementation research in various fields including nursing, quality and safety of care, infectious and inflammatory diseases, and business administration and change management. Interview notes were analysed using open coding and after which a codebook was developed (FN, AD). Codes were then linked to all interview summaries (Axial coding). Finally, selective coding was done to create 11 overarching domains which were discussed in the steering group for final verification. This formed the starting point for the first round of the e-Delphi study.

### e-Delphi

In the e-Delphi study, the implementation research community in the Netherlands was asked to confirm, refute, prioritise, and interpret the initial domains derived from initial interviews. The e-Delphi study consisted of two consecutive rounds to reach consensus.

#### Round 1

The first e-Delphi round was intended to collect suggestions from implementation researchers for relevant research topics within the domains. Using the same inclusion criteria as for the recruitment of interviewees during preparation, implementation researchers in the Netherlands were recruited through the NIC network. Participants were also able to suggest other names as well (snowball sampling). In total, 47 researchers were approached of which 26 (55%) participated in the first round.

Participants were asked to submit a maximum of 10 research questions online via Survalyzer and classify them in one of the 11 domains that were identified in the preparation (see Additional file 1). The first round was open for 4 weeks and a total of 200 research questions were submitted. The research questions were thematically analysed (FN, AD), giving all research questions a topic code, definition, and explanatory notes. Based on the submitted research questions, the initial domains were transformed into 7 themes covering 31 unique research topics. These were: determinants (barriers and facilitators), the process of and methods for matching implementation strategies to determinants, implementation strategies, implementation outcomes, measurement tools, research designs, and theories, models, and frameworks.

#### Round 2

In the second e-Delphi round, the list of research topics was refined and ranked based on consensus amongst particpants (see Additional file 2). Because the focus was on reaching consensus in this round and not on generating ideas, we only invited Round 1 participants for the second round.

Per research topic, participants were asked to indicate whether the topic needed to be integrated into the final research agenda using a five-point Likert-scale (1 = totally disagree to 5 = totally agree). Participants were also asked to indicate whether the definition and description of the topic was clear and comprehensive, using a five-point Likert-scale (1 = totally disagree to 5 = totally agree). Consensus to include a research topic in the research agenda was reached when 67% of the participants or more scored a topic 4 (agree) or 5 (totally agree) [32]. When consensus for including a particular topic was not reached, topics were merged, reformulated, or deleted from the list using the justifications provided by the participants.

### Survey: input from practitioners in the field

Practitioners working in healthcare in the Netherlands were invited to share their experiences in an online survey distributed via Survalyzer (see Additional file 3). They were asked to report a maximum of 10 knowledge gaps, reported in open-ended text fields. The survey was open to all practitioners working in healthcare and involved in implementing evidence-based interventions into routine practice in November 2021 – March 2022. Respondents were recruited through direct mailings and through the personal social media (LinkedIn, Twitter) pages of the research team members. In addition, various relevant organisations were approached to distribute the survey to their members or employees, including through newsletters by ZonMw, NIC, Amsterdam Public Health research institute, the KWF Dutch Cancer Society, and other similar organizations. Using the same method as before, the responses from the survey were coded using the topics from the e-Delphi study. To ensure practical utility of the resulting research agenda, the topics mentioned by practitioners were retained when they were also mentioned by implementation researchers, regardless if consensus was achieved in this group. New topics mentioned by practitioners were kept separate from the topics resulting from the e-Delphi.

### Data triangulation

Interview data were used to inform the 11 overarching domains that formed the starting point for the first round of the e-Delphi study. After round 1, these were transformed into 7 themes covering 31 unique research topics that were refined and ranked based on consensus amongst participants in round 2. Next, FN and AD coded the practitioner survey data in Excel against the Delphi research topics (both with and without consensus), and open coded the final survey data into additional topics.

## Results

### Sample

#### Researchers

In total, 47 implementation researchers in the Netherlands were approached for the e-Delphi of which 26 (55%) participated in the first round. Reasons for not participating in the e-Delphi study included conflicting priorities and excessive workload during the study period. In total, 73% of the participants were female and the majority had either six to ten years work experience (31%) or over 15 years (27%). Participants were active in a wide variety of health research fields, including Quality of Care (26%), Life Sciences & Health (12%), Prevention (12%), Youth Care (9%), Elderly Care (5%), and Mental Health (5%). Half (54%) had published or were involved in publications about implementation research more than 10 times. Of the 26 participants from Round 1, 22 participants (85%) also completed the second round. The main reasons for not participating were related to excessive workload and conflicting priorities.

#### Practitioners involved in implementation

In total, 74 practitioners filled out the survey. The majority had over 15 years of work experience (30%). A quarter (25%) worked as a researcher, 17% as a project or program manager, and 15% as an implementation specialist or advisor. Participants were active in a wide variety of fields, including Elderly Care (21%), Quality of Care (17%), and Prevention (17%).

### Implementation research agenda

**Fig. 2:**
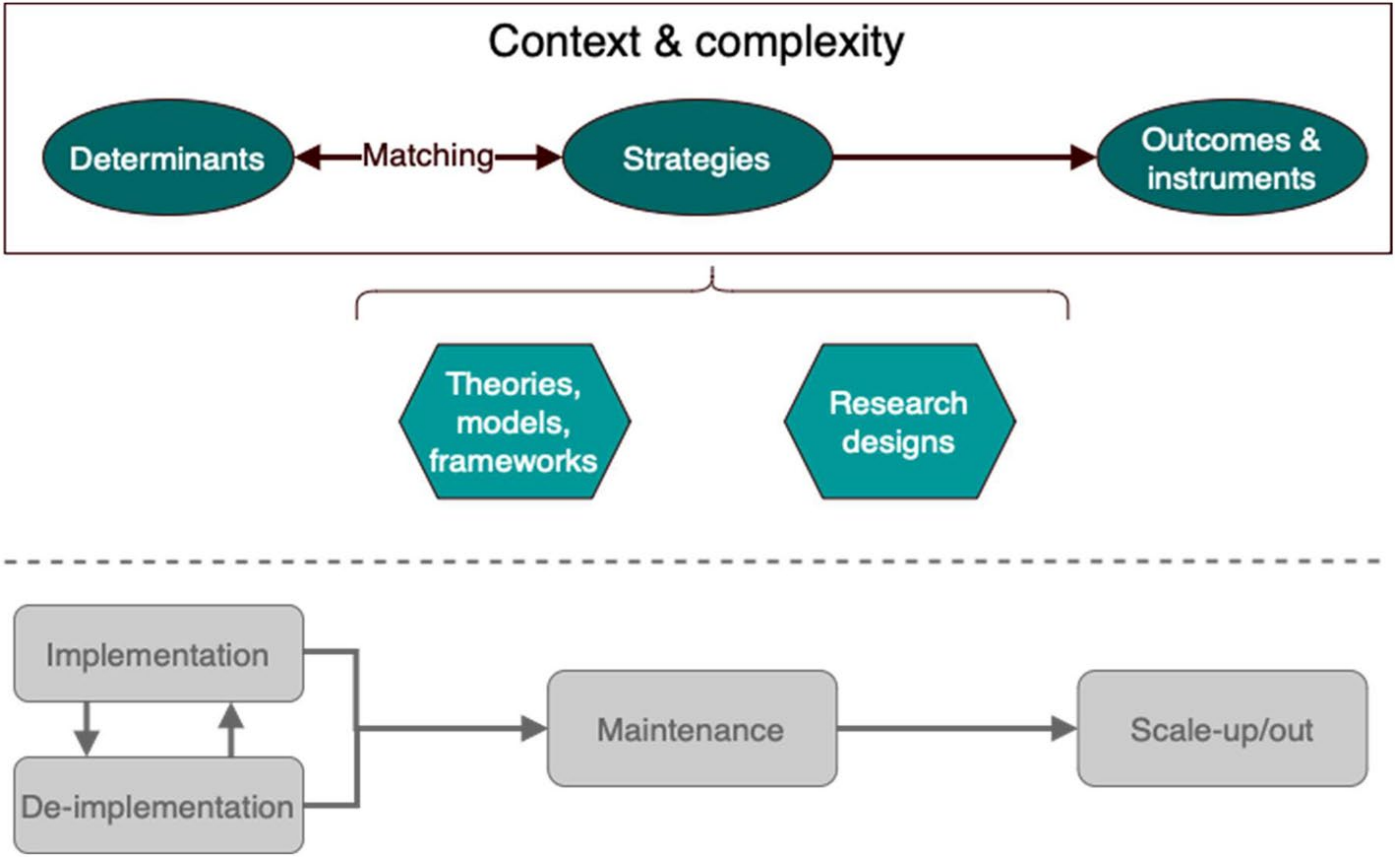
Overview of the main themes of the implementation research agenda. The bottom part below the dashed line is to illustrate the different phases of implementation

The 12 topics that reached consensus among researchers were categorised in six themes: 1) Determinants, 2) Methods for matching determinants to strategies, 3) Implementation strategies, 4) Outcomes and measurement instruments, 5) Theory, models, and frameworks, and 6) Research designs. Many of the themes are interlinked and can be related to one or more phases of implementation, e.g., research on determinants is relevant for implementation, de-implementation, sustainability, and scale-up. The research agenda is schematically depicted in Fig. 2 and detailed in Table 1.

**Table 1:** Themes and topics of the Dutch implementation research agenda noted by researchers (consensus-based) and by implementation practitioners (survey-based)

**Themes & topics**		**Description**	**Research**	**Practice**
***1. Determinants***				
	Analysing determinants	Methods for objective and critical reflection in identifying determinants of implementation.	-	V
	Determinant of interactions	Coherence (interaction, interdependencies) of determinants, their impact, and contextual influences.	V	-
	Determinants of (a) de-implementation, (b) scaling-up, and (c) sustainability	Identification and taxonomies of factors that determine de-implementation processes, scaling-up and sustained use, including the coherence of identified determinants.	V	V
***2. Matching strategies to determinants***				
	Matching strategies to determinants	Methods and insight in underlying mechanisms and complexity in linking implementation strategies to identified determinants and developing concrete implementation activities that are feasible, cost-effective, and acceptable.	V	V
***3. Implementation strategies***				
	Designs for testing strategies	Research designs for developing and testing the short and long-term effectiveness of strategies and the interactions between different multi-faceted strategies.	V	V
	Order of strategies	More knowledge about in what order strategies should be deployed and which approach is best for mapping the complexity of sequencing strategies.	-	V
	Working functions of strategies	Research on effective elements and effectiveness of single strategies, as well as multi-faceted strategies, incorporating the influence of context on the effectiveness of (active elements) of strategies.	V	V
	Working mechanisms of adaptation	Development and testing of methods, concepts, conditions, and working mechanisms of adapting implementation strategy to a specific context.	V	V
	Multi-level strategies	Explore interactions and alignment of strategies at different levels of the system (e.g., individual, within and between organisations, etc.).	V	V
	Strategies for (a) de-implementation, (b) scaling-up, or (c) sustainability	Research on the development and effectiveness of discrete, multi-faceted and blended strategies for de-implementation, scaling-up and sustained use in practice. Note that there was no consensus (by researchers) reached for topics b and c.	(V)	V
	Cost-effectiveness of strategies	Costs for implementation and cost-effectiveness or cost-benefit analysis of an implementation strategy.	V	-
***4. Implementation outcomes***				
	Measuring implementation outcomes	Guidance in measuring and comparing implementation relevant outcomes. This also relates to combining quantitative and qualitative evaluation methods.	-	V
	Measurement instruments	Availability and knowledge of valid and reliable measurement tools for strategies, implementation processes, and outcomes and Dutch-language versions of existing measurement tools.	-	V
***5. Theories, models, and frameworks***				
	De-implementation	Conceptualising and theorising mechanisms of action of de-implementation, such as cognitive bias, culture, stopping altogether, and/or substitution.	V	-
	Synthesis of theories, models, and frameworks	Guidance in selecting and utilising implementation theories, models, and frameworks.	-	V
	Inclusivity	Developing frameworks and methods for identifying, measuring, and addressing issues of inclusivity and equity in implementation, such as including hard-to-reach groups, literacy, and socio-economic inequalities.	V	V
***6. Research designs***				
	Use of routinely collected data	Research into methods for using routine data to monitor implementation processes and evaluate effectiveness of implementation strategies.	V	V
	Analysing implementation data	Knowledge on the use of digital tools and real-life data (from patient records or healthcare facility data) in actual implementation and evaluation thereof.	-	V
	Knowledge of designs	Knowledge and guidance in designing implementation evaluations, including methodological rationales, analytical tools, and reporting guidelines.	-	V
	Designs with multiple settings	Knowledge on how to aggregate results at the local level to a higher level to establish more generally applicable principles, keeping in mind the balance between practice dynamics and research quality.	-	V

### Theme 1: determinants

#### Analysing determinants

Methods for identifying and analysing determinants was indicated to be relevant to practitioners. Respondents felt that when obtaining information about potential facilitators and barriers for implementation, often, the more enthusiastic professionals contribute, potentially hindering insight into motives of the non-responders. In addition, practitioners raised the issue of power structures and power differences and the extent to which they influence the implementation of interventions. Researchers did not reach a consensus on this topic. They indicated during interviews that there is a considerable body of research on methods for identifying and analysing determinants available in literature.

#### Determinants of interactions

Research participants expressed that generalised knowledge about determinants of implementation practice is relatively saturated and multiple frameworks are available, such as in the Consolidated Framework for Implementation Research (CFIR, [33, 34]), Flottorp’s Tailored Implementation for Chronic Diseases (TICD) framework [35], and Fleuren’s MIDI (Meetinstrument voor Determinanten van Innovaties) [36]. Such frameworks provide taxonomies of various determinants and group them into pre-defined domains. However, current knowledge provides little insight in the interactions and independencies of determinants and the direction (positive or negative) or size (large or small) of impact they empirically have on implementation processes. Therefore, research on the coherence (interaction and/or interdependencies) of determinants or groups of determinants, their significance to implementation processes and outcomes, and possible contextual interrelatedness is required.

#### Determinants of (a) de-implementation, (b) scaling-up, and (c) sustainability

Both research and practice participants expressed that frameworks and taxonomies of determinants are lacking information on de-implementation, scaling-up, and sustainability. Regarding de-implementation, it is often assumed that the same factors determine de-implementation processes as they influence implementation processes. However, it is unclear if this indeed is the case and whether de-implementation is determined by specific determinants. Practice participants also raised the issue that they are often not aware that implementation and de-implementation are likely to be similar processes, but are influenced by different determinants and thus likely to require different strategies.

Regarding determinants for scaling-up interventions to increase population level impact, practitioners indicated that often, scaling-up follows successful implementation at smaller scale and reaching impact on different levels (i.e. between organisations, regional, national, or international) often takes more time and is not well documented. As a result, there is limited knowledge of the factors that determine successful intervention scaling-up. In addition, the criteria or conditions under which stakeholders decide to scale-up are unclear.

Research and practice participants also indicated that insight in determinants for sustaining a successfully implemented intervention in practice are lacking. Where many implementation projects have a clear start- and endpoint, sustainability concerns the phase when an intervention is normal part of practice and thus has no endpoint. More research is needed for gaining insight into the factors that shape and influence sustainment of interventions in practice. In addition, practice participants expressed a need for knowing what determinants are important to address in an early implementation phases to support and stimulate sustainability later.

### Theme 2. Matching determinants to strategies

#### Matching determinants to strategies

Both research and practice participants agreed that there is a knowledge gap regarding methods for matching implementation strategies to determinants. Research into methods for identifying, prioritising, and selecting implementation strategies that are known to be effective in addressing selected determinants is warranted. This was not only mentioned for implementation processes, but also for de-implementation, scaling-up, and sustained use of interventions in practice. From a practice perspective, selecting strategies is often a ‘black box’. Furthermore, this knowledge gap has a latent issue related to the causal pathway, i.e., mechanisms by which an implementation strategy addresses a certain determinant. There is limited empirical evidence of the components of implementation strategies contributing to achieving certain outcomes and notably, the extent to which these components and outcomes are related to the identified barriers.

### Theme 3. Implementation strategies

#### Designs for testing strategies

A hallmark of applied research is that it takes place in the complex real world which limits the possibility to control for interactional effects between (sets) of strategies and external factors. Often Randomised Controlled Trials (RCT) in the traditional parallel group comparisons are not feasible in real world conditions. In addition, implementation processes often have a considerable time horizon. These aspects pose significant challenges to designing research that aim to empirically prove the effectiveness of discrete or multifaceted implementation strategies with acceptable certainty. Advanced study designs for controlling and managing confounding factors, innovative statistical methods, and methods for combining multiple data sources and outcome data are required to advance the study of implementation strategies. Advancing study designs was mentioned by both researchers and practice participants.

#### Order of strategies

It would be useful to explore in what order strategies should be deployed. This was mentioned by the practitioners only and relates to the interdependencies and interactions of determinants and strategies.

#### Working functions of strategies

Both researchers and practitioners raised the issue of developing and testing causal pathways and the specific active elements within strategies, the interactions and order of delivery of multi-faceted strategies, and variations in effectiveness across contexts. A critical aspect of this research would be to identify and report the core working functions of implementation strategies that logically and empirically cause the change that can be attributed to the implementation strategy.

#### Working mechanisms of adaptation

Both researchers and practitioners also raised the issue of methods for adapting or tailoring implementation strategies to (local) determinants and contextual factors and develop actionable work plans. The empirical effectiveness and efficiency of methods for developing tailored implementation strategies should be explored.

#### Multi-level strategies

Determinants can influence implementation processes at different levels of the healthcare system. These include the individual (patients, professionals) level, levels within organisations, and levels between organisations within the system and its policies. The interplay of determinants and strategies at different levels is largely unknown. Both researchers and practitioners indicated this topic to be of interest to better understand interactions and being able to improve alignment of multiple strategies acting at different levels of the healthcare system and address potential issues of ownership, roles, and responsibilities.

#### Strategies for (a) de-implementation, (b) scaling-up, or (c) sustainability

Although considerable (descriptive) research has focussed on strategies for implementing evidence-based practices, participants expressed that less attention has been given to de-implementing or replacing ineffective or inefficient practices. Similarly, limited research has been devoted to developing and providing empirical proof of strategies for scaling-up as well as ensuring sustained use of successfully implemented interventions. Both researchers (for de-implementation) and practitioners (for de-implementation, scaling-up, and sustainability) highlighted this omission.

#### Cost-effectiveness of strategies

Besides empirical evidence of the causal effects of implementation strategies in addressing a determinant, the costs at which the effects are achieved are also relevant to consider in selecting and prioritising implementation strategies. Researchers reached consensus on the issue that there is limited knowledge of the cost-effectiveness of implementation strategies.

### Theme 4. Implementation outcomes

#### Measuring implementation outcomes

Although raised as an important research area in various international publications, the research community in the Netherlands did not agree that reliability and accuracy of implementation outcome measurement and interpretation should be a research priority. There was consensus on the research description (68%) but not on the definition of the topic (64%). This includes topics such as what to measure, operationalising measurement of determinants, quality and process indicators, and implementation success. Practitioners indicated that guidance is required on selecting, applying, interpreting, and comparing implementation outcomes. This also related to questions of defining implementation success in a particular setting or situation, and selecting valid, theory-supported indicators that can be measured reliably. These indicators can be both on the level of implementation strategies outcomes and process measures to monitor implementation progress.

#### Measurement instruments

There was no consensus among researchers (59%), but they did reach consensus on its definition (73%). According to practitioners, this topic should concern the development of valid and reliable measurement instruments (e.g., questionnaires) for determinants, implementation processes and outcomes and the further development or translation to the Dutch context of existing measuring instruments.

### Theme 5. Theory, models, and frameworks

#### De-implementation

Researchers mentioned that theories, models, and frameworks around de-implementation are a topic of interest and are perceived to have received, until now, relatively little attention. Conceptualising and theorising mechanisms of action of de-implementation is required to verify and explain differences between stopping with certain practices (de-implementation) and starting with new practices (implementation). Research is needed to understand processes of de-implementation, its determinants, contextual aspects, as well as strategies to improve outcomes, and implementation measures.

#### Synthesis of theories, models, and frameworks

There are many theories, models and frameworks that can help guide, explain, or evaluate implementation processes available [3]. The fact that these theories, models, and frameworks co-exist and there is no, or not yet, one approach that aims do it all, is a virtue of practice and the Dutch community of researchers. There are no dominant schools of thought, rather, because of the shear complexity, dynamic nature and contextual dependencies, all efforts that systematically aim to contribute to understanding implementation as an object of study are welcomed. The downside, as expressed by practice, is that it is difficult to select and apply a theory, model, or framework in practice. Theories, models, and frameworks have limitations and specific purposes which are not always clear or difficult to translate to the practical situations at hand [3]. Indeed, practitioners indicated that there is a lack of scientifically grounded guidance in selecting, adapting, and using existing implementation theories, models, and frameworks.

#### Inclusivity

From the research perspective, it relates to including and involving stakeholders (i.e., people) from different genders, age, ethnicities, educational background, employment, socio-economic status, etc. when relevant to the implementation research that is conducted. Insight into participants' representativeness in quantitative and qualitative research designs is fundamental to the generalisability of findings. In addition, the impact of underrepresentation of certain groups on implementation processes or outcomes is unclear. From a practice perspective, the issue of inclusivity and equity relates to extending identifying determinants and strategies with various stakeholders with different backgrounds.

### Theme 6. Research designs

#### Use of routine collected data

Related to the issue of research designs, both researchers and practitioners indicated that the lack of using routine data for implementation insights should be prioritised. In current healthcare organisations and research projects a large amount of data is collected, the quality is often not sufficient for high quality analyses. To reduce research waste and lower the burden on both participants and researchers, novel methods are required to enable using existing data for evaluating and monitoring implementation processes. To illustrate, researchers suggestions were interrupted time series, using AI or big data methods.

#### Analysing implementation data

Related to the previous topic, practitioners also raised the issue of using digital tools and real-life data (from patient records or healthcare facility data) in implementation and evaluation of implementation.

#### Knowledge of designs

Practitioners indicated that like utilising implementation theories and frameworks, guidance and proven methods are required in designing the evaluation of an implementation project. This includes designing and conducting process evaluations in a structured way.

#### Designs with multiple settings

The applied nature of implementation research can also have implications for generalisability of findings to other interventions, organisations, or settings. Practitioners indicated that there is a need for knowledge on aggregating results at the local level to a higher level to establish more generally applicable principles, keeping in mind the balance between practice dynamics, research quality and possible trade-offs that are made to the certainty of conclusions.

### Additional implementation practice topics

Complementary to the topics listed in Table 1, practitioners listed several additional research areas relevant to implementation. These topics did not converge directly with the topics that were included in the e-Delphi. They are summarised in Table 2. In general, practitioners’ responses often included references to the need for ‘*how to*’ tools and guidelines as well as capacity building in terms of training and education of practitioners. Related to this is having access to trained implementation researchers, which highlights the importance of a network so that practice and research communities can find each other and collaborate. Furthermore, implementation practice often extends periods of research. Funding to contribute to measuring longer-term effectiveness of implementation strategies or issues of sustainability and scaling-up should receive attention and subsequent funding.

**Table 2:** Additional knowledge requirements mentioned by implementation practitioners

Themes & topics		Description
***Need for trained practitioners, implementation specialists, researchers***		
	Basic implementation knowledge	There is often a lack of basic knowledge about implementation, such as when to start an implementation process, selecting and developing a strategy, involving stakeholders, use of frameworks, measuring outcomes, etc. Related to this is a need for formal accreditation or competency profile that provides insight into which expertise implementation specialists need and which training pathways are suitable for this. In addition, people with implementation (research) expertise within the organisation are not always found or they simply are not present
***Lack of time and capacity***		
	Capacity	In response to the limited available basic implementation knowledge, capacity needs to be increased. There are too few people with enough implementation expertise, leaving too little capacity for implementation guidance in practice, but also for implementation specialists when applying for grants. Including implementation (research) in other training programs, refresher courses, and knowledge exchanges between experts could contribute to this
	Staff turnover	Changes in personnel (i.e., implementation, policy, and/or management) pose a major problem for implementation as this is often a longer-term endeavour. More knowledge is needed for mitigating measures to limit impact of staff turnover on implementation and sustainment
***Need for roadmaps, tools, checklists***		
	Development and synthesis of roadmaps, tools, etc	Translation of scientific knowledge into practical tools such as a toolbox of implementation strategies, tailor implementation strategies organisation-specific, tools for determining implementation readiness, guidance for evaluation, and/or tools for choosing strategies, models, and for mapping interdependencies of determinants
	Effectiveness of existing roadmaps, tools, etc	Proof about the effectiveness and usability of existing roadmaps, tools, and guidance for implementation practice
***Funding***		
	Time between pilot and sustainment	Knowledge of ways for bridging the time between ending effective pilot project and achieving successful sustainment with structural funding of the intervention
	Structural funding	Knowledge about funding (streams) of implementation, sustainment, and dissemination. Mostly, funding is focussed on new research and less for the further roll-out of proven effective interventions. In addition, funding for healthcare organisations often does not contain the right funding incentives for implementation and sustainment. This would require longer-term vision and commitment on innovation in healthcare settings
***Other issues***		
	Ownership	Knowledge about shaping ownership and associated responsibilities of interventions and implementation processes. Researchers often start thinking about this too late (i.e. after their research project is finished). In addition, expectations are not always properly expressed to and by stakeholders
	Endorsement	Knowledge and tools of how to create and maintain support (of end-users, professionals, policymakers, and management) at different layers of involved and affected stakeholders
	Digitalisation	Knowledge on implementation of digital interventions and their embedding and relatedness to existing IT systems, such as electronic health records
	Interprofessional collaboration	Knowledge about interprofessional cooperation in implementation, its preconditions and methods for engagement
	Knowledge dissemination	Knowledge on how to ensure that knowledge products are disseminated rather than just posted on a website. This also relates to the issue of ownership and (sustained) funding

## Discussion

This paper presents potential research topics for implementation of evidence-based practices in the Dutch healthcare setting. It is the first agenda to combine perspectives from both the implementation science and implementation practice communities. The agenda outlines priorities for implementation research, focusing on methods for matching strategies to determinants, understanding the mechanisms and pathways through which implementation strategies operate, measuring implementation outcomes, promoting inclusiveness in implementation research, advancing research designs for testing implementation strategies, developing de-implementation strategies and creating multi-level strategies. Furthermore, there is a need for guidance in designing evaluation studies that enable reliable and feasible evaluation of implementation processes and outcomes.

The methodology used in this study is more rigorous compared to earlier work which often relied on the personal experiences and perspectives of the authors. Nevertheless, many topics identified in this study are similar to those found in international agendas [1, 5–20].

This study highlights a shift from merely identifying determinants to focusing on the mechanisms and effectiveness of implementation strategies and paying attention to the dynamics in different phases of implementation work (pre-, implementation, sustainment, scaling, de-implementation). Advancing methods for matching implementation strategies to determinants, developing valid process and outcome measurement instruments, and addressing the complexity of context in practice are crucial areas for the future of the field [37]. While much of the focus in implementation research has been at the individual and organisational level, there is growing recognition of the need to address system-level dynamics.

From practitioners' perspective, integrating and embedding new evidence-based practices (or discontinuing practices) is not solely an issue of implementation or implementation science.

While implementation science provides theory-informed frameworks for understanding determinants, strategies, and outcomes of practice change [3], practitioners routinely draw on organizational change management to address leadership engagement, stakeholder buy-in, and the emotional and cognitive processes that influence change. Quality improvement approaches, such as Plan–Do–Study–Act cycles, offer structured, data-driven processes for iterative testing and integration of new practices into routine workflows [38]. Likewise, traditions of knowledge utilization and knowledge transfer emphasize how research evidence is accessed, interpreted, and adapted within local contexts, highlighting the social and organizational processes that shape knowledge movement and use [39–41]. Collectively, these perspectives demonstrate that practice change is not solely a technical implementation problem, but a multidimensional socio-organizational process. Consequently, effective implementation of new practices in real-world settings typically is inherently transdisciplinary, non-linear, and dynamic, shaped by the medical, social, and behavioural contexts in which it takes place.

This agenda builds on existing views of research priorities by explicitly incorporating the practice perspective. Although generating scientific knowledge is valuable in its own, science should ultimately serve practice. Despite the growing methodologies for harnessing practice experiences into intervention development such as action research, perspectives of the science community in implementation science are still often prioritised over those of practitioners [42]. Whereas generating generalizable knowledge of implementation methods and processeses is at the core of scientific enquiry, the practical utility of this knowledge is to be warranted to ultimately improve healthcare practice. Balancing scientific inquiry with implementation practice is crucial to prevent creating a new research-practice gap; the very object of and reason for implementation science to exist as a discipline in the first place.

### Strengths and limitations

In this study, a multi-method structured approach was employed to involve a range of stakeholders and triangulate perspectives from both implementation researchers and practitioners. Another strength of the study is that implementation researchers and practitioners from all healthcare domains in the Netherlands were asked to share their ideas on important topics for the research agenda. While this approach generated a broad and comprehensive list of relevant topics, resulting in a list of potential research topics, it also means that some topics may lack the specific details relevant to certain settings, fields, or types of interventions. As this is the first agenda in the Netherlands, participants were recruited from the healthcare context in the Netherlands, therefore some topics may have received more attention due to their particular relevance to the Netherlands. The contextual features of the Dutch healthcare and implementation research context are likely to have shaped the agenda-setting by emphasising pragmatic and practice driven research topics. International readers should interpret these findings in light of differences in funding centralisation, governance structures, and healthcare capacity, as such system-level factors strongly influence the feasibility and transferability of implementation research priorities to other settings. Nonetheless, the alignment of these findings with other international research agendas suggests that they may also be applicable to healthcare settings in other countries.

There are also methodological limitations that should be acknowledged. The combination of the e-Delphi and survey proved effective for engaging both researchers and practitioners in the Netherlands and for reaching consensus. However, the number of participants in each method was relatively small. This could partly be attributed to the size of the Netherlands, where there are relevatively few academics who explicitly identify as implementation researchers. Many researchers may have an interest in implementation science, but it is not always their primary focus or field of research. Furthermore, many researchers take on multiple roles, often participating in or leading practical implementation projects. In these roles, their focus may be more on improving the uptake of evidence-based interventions in practice rather than on developing generalisable scientific knowledge.

### Recommendations for using the research agenda

Our primary recommendation is to build on existing knowledge and prioritise the topics outlined in this agenda. We stress the importance of conducting experimental and empirical research to better understand and predict causal patterns involved in the implemention and de-implemention of complex interventions in practice. Detailed reporting of implementation strategies and evaluations designs is essential, with a focus on core functions and causal pathways. Advanced, practice-centered, study designs should also be considered. Examples of such methodological advancements include hybrid effectiveness–implementation designs [43], adaptive designs like Sequential, Multiple Assignment Randomized Trials (SMART, [44]) and Multiphase Optimization Strategy (MOST, [45]), or learning health system and embedded research designs that integrate continuous data collection, rapid feedback loops, and co-learning between researchers and practitioners [46]. Such research designs have the potential to transform routine practice settings into ongoing research environments enabling continuous learning. Additionally, it is crucial to translate research findings into pragmatic tools such as the D&I Models Webtool [47], the ItFits-toolkit [48] and Map2Adapt [49], that can help practitioners select and operationalise strategies into concrete, actionable workplans tailored to local needs and requirements.

This research agenda is not intended to be a fixed guideline rather than a reflection of current priorities. It should be regularly updated and refined by the field, taking into account both national and international developments as well as progress made on the priorities identified in this agenda.

## Conclusions

Research into the complexity of implementation processes and methods is advancing both nationally and internationally. Implementation researchers and practitioners in the Netherlands have identified several key topics that should be prioritised to enhance the current state of knowledge and improve implementation practices. These priorities include matching strategies to determinants, understanding the mechanisms and causal pathways of implementation strategies, measuring implementation outcomes, promoting inclusiveness, and advancing research designs for developing and testing (de-)implementation strategies. Additionally, there is a clear need for practical tools and guidance to design evaluation methodologies that ensure reliable and feasible assessment of implementation processes and outcomes. This agenda provides researchers, practitioners, policy makers and funders in the Netherlands and beyond with clear directions and encourages them to use it as a framework to align their focus and priorities in implementation research.

## Supplementary Information

Additional file 1: Domains of first round of the e-Delphi study.

Additional file 2: Items of second round of the e-Delphi study.

Additional file 3: Survey for implementation practice.

## Data Availability

The datasets used and/or analysed during the current study are available from the corresponding author on reasonable request.

## Abbreviations

NIC: Netherlands Implementation Collective
CFIR: Consolidated Framework for Implementation Research
RCT: Randomised Controlled Trials
