# Supplementary material for: Topics for implementation research: Implementation researchers’ and practitioners’ views in The Netherlands
Source: Implement Sci Commun. 2026 Feb 24;7:63. doi: 10.1186/s43058-026-00890-6 (PMC13041016; doi:10.1186/s43058-026-00890-6)
Supplement: Supplementary file 1 — Additional file 1: Domains of first round of the e-Delphi study. [file 43058_2026_890_MOESM1_ESM.docx]

**Additional file 1: Domains of first round of the e-Delphi study**

**Implementation Research Domains**

To gain an initial understanding of knowledge gaps in implementation research, 19 interviews were conducted with implementation researchers in the Netherlands, and a document analysis was carried out. This document presents the results of these analyses, organized into domains. Within each domain, implementation researchers identified knowledge gaps that the research agenda should focus on. Each domain is briefly explained, and the domains are listed in no particular order of importance. Additionally, there is an ‘Other’ domain. This document can be used to categorize research questions during submission.

**Domain 1: Implementation Research Designs**
The "Implementation Research Designs" domain refers to research on the application and further development of new designs, such as action research, living labs, innovation journeys, and the use of big data.

**Domain 2: Frameworks/Models**
The "Frameworks/Models" domain involves deeper research into, for example, the effectiveness of certain frameworks/models, as well as further theorizing or applying these frameworks/models.

**Domain 3: Measurement Instruments**
The "Measurement Instruments" domain refers to all methodological aspects applicable to implementation research. This could include comparing different measurement tools or methods, developing new instruments, and exploring how to best measure implementation.

**Domain 4: Context**
The "Context" domain refers to the need for more research on standardizing, influencing, and accounting for contextual factors in implementation processes.

**Domain 5: Systems Approach**
The "Systems Approach" domain involves viewing implementation within a larger system, where there is interaction between different dynamic systems. This could include research on how organizational hierarchy affects implementation.

**Domain 6: Determinants**
The "Determinants" domain refers to further theoretical development of certain determinants or research on how to identify which determinants are most influential in implementation processes.

**Domain 7: Implementation Strategies**
The "Implementation Strategies" domain focuses on research into the selection, evaluation, and application of strategies in different contexts.

**Domain 8: Adaptation**
The "Adaptation" domain involves research into the degree of flexibility that can be applied during implementation, while still maintaining evidence-based practices.

**Domain 9: De-implementation**
The "De-implementation" domain refers to further research into the relationship between implementation and de-implementation, as well as a deeper investigation into de-implementation processes in general.

**Domain 10: Scaling Up**
The "Scaling Up" domain highlights the need for more research on the effective elements for scaling up, how these processes unfold, and how to research them effectively.

**Domain 11: Other**
Additionally, there are various other research topics that could contribute to advancing the field of implementation science. These are categorized under the "Other" domain, as they were not mentioned by many respondents. Examples include sustainability, the impact of digitalization on implementation processes, economic evaluation of implementation, and inequality within implementation.
