# Supplementary material for: Topics for implementation research: Implementation researchers’ and practitioners’ views in The Netherlands
Source: Implement Sci Commun. 2026 Feb 24;7:63. doi: 10.1186/s43058-026-00890-6 (PMC13041016; doi:10.1186/s43058-026-00890-6)
Supplement: Supplementary file 2 — Additional file 2: Items of second round of the e-Delphi study. [file 43058_2026_890_MOESM2_ESM.docx]

**Additional file 2: Items of second round of the e-Delphi study**

**Thematic List Delphi Round 2**

In Delphi Round 1, implementation researchers in the Netherlands identified topics they believe require further investigation. The submitted topics were grouped into themes and specific topics. The table below presents the themes, topics, and descriptions that will be evaluated in Round 2.

| **Theme** | **Topics** | **This topic includes...** |
| --- | --- | --- |
| **1. Determinants** | **1.1 Determinant Analysis** | Research on how to best conduct a determinant analysis and whether different methods (e.g., surveys or interviews) yield different determinants. Additionally, how to select the most important determinants. |
|  | **1.2 Order of Determinants** | Research on the importance of the order in which determinants are addressed. |
|  | **1.3 Interaction Between Determinants** | Research on the dependency and independence of determinants, and the context in which they exist. |
|  | **1.4 Determinants of De-implementation** | Research on the determinants of de-implementation and how they differ from the determinants of innovation implementation. |
|  | **1.5 Determinants of Scaling Up** | Research on the determinants involved in scaling up innovations. |
|  | **1.6 Determinants of Sustainability** | Research on the determinants for sustaining innovations. |
|  | **1.7 Effectiveness of Determinants** | Research on testing the effectiveness of individual determinants, including the interaction between different determinants. |
| **2. Linking Determinants to Implementation Strategies** | **2.1 Linking Determinants to Implementation Strategies** | Research on how identified determinants can be linked to implementation strategies, with a focus on understanding the causality between determinants and strategies. |
| **3. Implementation Strategies (referred to as strategies)** | **3.1 Designs for Testing Strategies** | Research on new designs for testing the effectiveness of strategies, including both short- and long-term effects, as well as the interaction between various multi-faceted strategies deployed in parallel. |
|  | **3.2 Order of Strategies** | Research on the order in which strategies should be implemented and the appropriate research designs to test this order. |
|  | **3.3 Effective Elements of Strategies** | Research on the effective elements and effectiveness of individual strategies, as well as multi-faceted strategies, with consideration of contextual differences. |
|  | **3.4 Mechanisms of Adaptation** | Research on methods, concepts, and mechanisms of adapting strategies (tailoring), including how to apply this systematically and the role of context. |
|  | **3.5 Effectiveness of Adaptation** | Research on the effectiveness of tailoring strategies, and whether each strategy should be tailored. |
|  | **3.6 Cost-effectiveness of Strategies** | Research on the cost-effectiveness of one or more strategies. |
|  | **3.7 De-implementation Strategies** | Research on specific strategies for de-implementation and their effectiveness. |
|  | **3.8 Strategies at Different Levels of the System** | Research on the interaction and coordination of strategies at various levels of the system (e.g., different departments, locations, etc.). |
| **4. Implementation Outcomes** | **4.1 Measuring Implementation Outcomes** | Research on how best to measure implementation scientifically, particularly focusing on what should be measured (e.g., operationalization of determinants, quality of implementation, implementation success, and exposure). Distinction should be made between process and implementation outcome measures. |
|  | **4.2 Measuring Scaling Up** | Research on developing methods to measure and evaluate scaling-up processes. |
|  | **4.3 Measuring Sustainability** | Research on developing methods to measure and evaluate sustainability. |
| **5. Research Designs** | **5.1 Gaining Generalizable Knowledge** | Research on how generalizable knowledge can be gained from different research designs, such as living labs, hybrid designs, mixed methods, etc. |
|  | **5.2 Using Routine Data** | Research on the use of routine data to evaluate implementation processes and the effectiveness of implementation strategies, such as evaluating audit and feedback and decision support. |
|  | **5.3 Analyzing Implementation Data** | Research on methods and techniques for analyzing implementation data, such as applying Artificial Intelligence (AI), machine learning, big data, and modeling to gain insights into implementation processes and effectiveness. |
|  | **5.4 Designs Allowing for Adaptation** | Research on designs that allow for adaptation and flexibility within the complexity of implementation processes. |
| **6. Measurement Instruments** | **6.1 Measurement Instruments** | This topic involves the development of valid and reliable measurement instruments for determinants, implementation processes, and outcomes, as well as further developing or adapting existing instruments to the Dutch context. |
| **7. Theories, Frameworks, and Models** | **7.1 Place of Adaptation in Models** | Research to better understand how adaptation is integrated into existing theories, frameworks, and models. |
|  | **7.2 Testing Interaction Between Elements in Existing Theoretical Models** | This topic involves testing the various elements and concepts within theoretical models, and their interrelationships, with a systemic perspective. |
|  | **7.3 Applying Models** | Research in which findings from theoretical concepts are used to test the validity of models. |
|  | **7.4 Framework for De-implementation** | Research on generating theories about the mechanisms of de-implementation, including cognitive bias, culture, complete cessation, and/or substitution. |
|  | **7.5 Differences Between Implementation and De-implementation** | Research to clarify the differences and overlap between implementation and de-implementation in terms of determinants, strategies, outcomes, and measurement instruments. |
|  | **7.6 Synthesis of Models** | This topic concerns reducing and/or synthesizing models, such as through a synthesis of models, including theory from other disciplines. |
|  | **7.7 Inclusivity** | This topic describes frameworks that incorporate inclusivity in implementation, such as involving hard-to-reach groups in implementation research and addressing social inequalities. |
