# Supplementary material for: Topics for implementation research: Implementation researchers’ and practitioners’ views in The Netherlands
Source: Implement Sci Commun. 2026 Feb 24;7:63. doi: 10.1186/s43058-026-00890-6 (PMC13041016; doi:10.1186/s43058-026-00890-6)
Supplement: Supplementary file 3 — Additional file 3: Survey for implementation practice. [file 43058_2026_890_MOESM3_ESM.docx]

**Additional file 3: Survey for implementation practice**

**[Next Page]**

**Instructions**

I would like to ask you to describe up to 10 knowledge gaps in the survey that relate to the implementation of innovations/interventions/guidelines in the healthcare context in which you work. These should not be specific to a particular innovation/intervention/guideline, such as:

- "The implementation of my intervention X failed,"
  but rather broader, more abstract knowledge gaps, such as:
- "Creating support is difficult,"
- "Scaling interventions remains a challenge."

After indicating the knowledge gaps, you will be asked questions about your demographic data. Please note that your responses will only be submitted once you click "Submit" on the final page.

**[Section 2]**

**Part 1: Experienced Implementation Problems**

I would like to ask you to describe up to 10 knowledge gaps in the survey that relate to the implementation of innovations/interventions/guidelines/etc., in the healthcare context in which you work. You can also provide a brief explanation. You may submit up to 10 knowledge gaps.
We ask you to describe the issue as specifically as possible so that we can get a clear picture of the challenges you face based on the information you provide.

When you have finished submitting the knowledge gaps, click at the bottom of this page on [next step] to proceed to the last section of the survey.

**Knowledge Gap 1:**
<enter a knowledge gap related to implementation>

**Knowledge Gap 2:**
<enter a knowledge gap related to implementation>

**Knowledge Gap 3:**
<enter a knowledge gap related to implementation>

**Knowledge Gap 4:**
<enter a knowledge gap related to implementation>

**Knowledge Gap 5:**
<enter a knowledge gap related to implementation>

**Knowledge Gap 6:**
<enter a knowledge gap related to implementation>

**Knowledge Gap 7:**
<enter a knowledge gap related to implementation>

**Knowledge Gap 8:**
<enter a knowledge gap related to implementation>

**Knowledge Gap 9:**
<enter a knowledge gap related to implementation>

**Knowledge Gap 10:**
<enter a knowledge gap related to implementation>

**[Section 3]**

**Part 2: General Questions**

**2. What is your gender?**
<drop-down menu>

- Male
- Female
- I identify as...
- Prefer not to say

**3. Which organization(s) do you work for?**
<enter the organization(s) you work for>

**4. In which healthcare domains are you involved in implementation?**
<check all that apply>

- Sports and Exercise
- Prevention
- Palliative Care
- Elderly Care
- Life Sciences & Health
- Quality of Care
- Youth
- Health Protection
- Pharmaceuticals
- Disabilities and Chronic Illnesses
- Mental Health
- Other, namely...

**5. How many years of work experience do you have in this field?**
<drop-down menu>

- 0-5 years
- 6-10 years
- 11-15 years
- More than 15 years

**6. What is your job title?**
<check all that apply>

- Implementation Advisor/Specialist
- Quality Officer
- Project/Program Leader
- Lecturer
- Manager
- Consultant
- Researcher
- Policy Advisor
- Healthcare Professional
- Other, namely...

**7. Do you consider these implementation activities as part of your main job/function?**

- Yes, I consider implementation my primary task.
- No, I do not consider implementation my primary task.

**[Section 4]**

Thank you very much for participating in this survey. We truly appreciate you taking the time and sharing your expertise.
